# Flumatinib plus venetoclax as an effective therapy for Philadelphia chromosome‐positive acute myeloid leukemia: A case report

**DOI:** 10.1002/ccr3.6688

**Published:** 2023-01-03

**Authors:** Si‐Man Huang, Tao Tao, Chao‐Ling Wan, Tian‐Mei Wu, Han‐Yu Cao, Yan Qiu, Xiang‐Dong Shen, Bin‐Ru Wang, Shuai‐Shuai Ge, Yan‐Yan Li, Tong‐Tong Zhang, Bing Wu, Sheng‐Li Xue

**Affiliations:** ^1^ National Clinical Research Center for Hematologic Diseases, Jiangsu Institute of Hematology The First Affiliated Hospital of Soochow University Suzhou China; ^2^ Institute of Blood and Marrow Transplantation, Collaborative Innovation Center of Hematology Soochow University Suzhou China; ^3^ Department of Respiratory and Critical Medicine the Fifth People's Hospital of Suzhou Suzhou China; ^4^ Department of Respiratory and Critical Medicine the Affiliated Infectious Diseases Hospital of Soochow University Suzhou China; ^5^ Suzhou Hospital of Chinese Traditional Medicine the Affiliated Hospital of Nanjing University of Chinese Traditional Medicine Suzhou China

**Keywords:** flumatinib, Philadelphia chromosome‐positive acute myeloid leukemia, regimen, venetoclax

## Abstract

Philadelphia chromosome‐positive acute myeloid leukemia (Ph + AML) is a rare type of AML with a low survival rate and poor prognosis. We first report a Ph + AML patient who remained in long‐term remission after the combination of flumatinib and venetoclax, which could provide corresponding treatment ideas for clinical practice.

## Abstract 2

The figure shows the treatment process of the patient and the dynamic changes in blood cell count during venetoclax and flumatinib. The *BCR::ABL1* transcript was negative after two courses of the combination of venetoclax anf flumatinib. No serious adverse events occurred during the treatment, although hematological toxicity was observed.

## 1 INTRODUCTION

The Philadelphia chromosome, causing the *BCR::ABL1* fusion gene, is generated by the reciprocal translocation of the long arms of chromosomes 9 and 22. This translocation fuses sequences of the *BCR* gene on chromosome 22 with regions of *ABL1* on chromosome 9.^1^ The incidence of the Philadelphia chromosome is greater than 95% among patients with chronic myeloid leukemia (CML).^2^ The incidence among AML patients is as low as 0.5%–3%. It is classified in the poor‐risk category according to the European Leukemia Net recommendations.^3^, ^4^ Due to the low incidence of this type and few case reports, the treatment regimen of Ph + AML has not been uniformly determined.

Here, we report a female patient who did not achieve remission with the HA regimen (homoharringtonine 2 mg/d d1‐7, cytarabine 100 mg qd d1‐7) and suffered disease relapse twice after salvage by imatinib‐based therapy, under the premise that the patient could not tolerate hematopoietic stem cell transplantation (HSCT). Surprisingly, the patient achieved long‐term remission after receiving the treatment of flumatinib combined with venetoclax. Taking the above into account, we hold that it is an effective and safe regimen which could provide a new option for Ph + AML patients who are resistant to conventional chemotherapy.

## 2 CASE HISTORY

**FIGURE 1:**
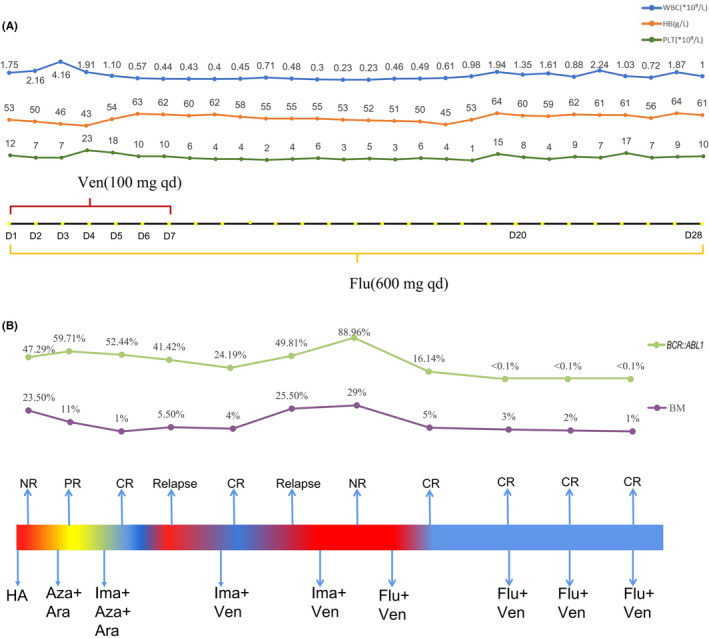
(A) Dynamic changes in blood cell count during ven + flu treatment. (B) Treatment process of the patient. CR, complete remission; PR, partial remission; NR, non‐remission; HA, homoharringtonine and cytarabine; Aza, 5‐azacitidine; Ara, cytarabine; Ima, imatinib; Ven, venetoclax; Flu, flumatinib; BM, bone marrow blast cell. *BCR::ABL1*, the presence of *BCR::ABL1* fusion gene calculated with standard materials was normalized with respect to the number of *ABL1* transcripts and expressed as copy numbers per 1 × 10^4^ copies of *abl*.

A fifty‐two‐year‐old female patient with right axillary masses was admitted to a local hospital, exhibiting an elevated level of leukocytes for 10 days. The patient was in poor physical condition with a history of modified radical mastectomy, undifferentiated connective tissue disease (UCTD), allergic rhinitis, and mycoplasma pneumonia. The blood count showed white blood cells of 31.57 × 10^9^/L, hemoglobin of 80 g/L and platelets of 90 × 10^9^/L. No hepatomegaly or splenomegaly was detected at diagnosis. Bone marrow (BM) morphology predicted acute myeloid leukemia. A peripheral blood smear showed 26% blasts. Cytogenetic analysis revealed a karyotype of 46,XX,t(9;22)(q34;q11)[20], and the RT–PCR analysis of *BCR::ABL1*(p210) was positive. A next‐generation sequencing‐based analysis for the detection of *ABL1* kinase mutation was negative. According to Chinese guidelines for the diagnosis and treatment of adult acute myeloid leukemia (not APL) (2021),^5^ the patient received the first induction therapy with the HA regimen. Then, the BM smear still showed 23.5% blasts. For further treatment, she went to the First Affiliated Hospital of Soochow University. She was in poor physical condition, and the Eastern Cooperative Oncology Group Performance Status scale (ECOG PS) score was 2.^6^ She was treated with 5‐azacitidine (100 mg qd d1‐7) and cytarabine (0.15 g q12h d1‐10). Subsequently, the BM showed 11% blasts. The flow cytometry (FCM) showed that the minimal residual disease (MRD) was 9.51%. Then, she was treated with imatinib (400 mg qd), 5‐azacitidine (100 mg qd d1‐7) and cytarabine (0.15 g q12h d1‐10). Then, the BM showed 1% blasts. The FCM showed that MRD was 0.18%. The RT–PCR analysis for the *BCR::ABL1*(p210) transcript was 52.44%. Due to significant myelosuppression, imatinib was reduced to discontinuation. However, the disease relapsed after drug withdrawal for 1 month. The FCM showed that MRD was 35.82%. The *BCR::ABL1*(p210) transcript was 41.42%. Subsequently, the patient was treated with imatinib (400 mg qd) combined with venetoclax (100 mg qd d1‐7) therapy and achieved complete remission (CR) after one course. The BM showed 4.0% blasts and the MRD was 0.019%. However, the disease recurred a month later with 25.5% blasts in the BM. After repeated treatment with venetoclax plus imatinib, the patient still showed non‐remission with 10% blasts in the BM and MRD was 42.3%. The *BCR::ABL1*(p210) transcript was elevated to 88.96%. At this time, the therapy was changed to venetoclax (100 mg qd d1‐7) combined with flumatinib (600 mg qd d1‐28). She achieved CRi (complete remission with incomplete count recovery) after one course of reinduction with 5% blasts in the BM and 0.22% MRD. Cytogenetic analysis revealed a karyotype of 46,XX,t(9;22)(q34;q11) [9]/46,XX [1]. Furthermore, the *BCR::ABL1* transcript was negative after two courses of therapy up to the time of manuscript submission. No serious adverse events occurred during the treatment, although hematological toxicity was observed (Figure 1A). In the subsequent consolidation therapy, the flumatinib plus venetoclax regimen was administered regularly 3 times (Figure 1B). The last FCM showed that MRD was 0.018%, and the patient had been in relapse‐free status for 7 months.

## 3 DISCUSSION

Before the tyrosine kinase inhibitor (TKI) was approved, Ph + AML patients were treated with traditional chemotherapy; However, the outcomes varied between cohorts. According to Cuneo A's report, 4 patients with Ph + AML received traditional chemotherapy, and all achieved CR. The median overall survival (OS) time was 7 months.^7^ However, another study showed that none of the 6 patients achieved remission.^8^ The advent of TKI changed the approach to Ph + AML. The study reported that 13 patients were treated with imatinib alone and the median OS time was 18.5 months.^9^ G.J. Min reported that 10 patients were treated with traditional chemotherapy combined with imatinib, and the median OS time was 6.5 months.^10^ To the best of our knowledge, the combination of flumatinib and venetoclax has not been reported in clinical application.

Venetoclax is a selective oral inhibitor of BCL‐2 which plays an important role in the treatment of hematological malignancies. In 2021, venetoclax in combination with hypomethylation agents or cytarabine was approved by the Food and Drug Administration (FDA) for the treatment of newly diagnosed elderly AML patients deemed ineligible for intensive therapy. Since then, clinical studies based on venetoclax have gradually been conducted to explore better treatment options. One of these new explorations is adding venetoclax to the therapy of Ph + AML. A. Maiti conducted a retrospective study in patients with Ph + AML (*n* = 7) who received venetoclax and TKI combined with chemotherapy regimens. The complete remission rate (CRR) was 28.6% and the objective response rate (ORR) was 43%. The median OS time was 2 months. The median relapse‐free survival time was 3.6 months.^11^ Ph + AML appears to be an aggressive disease and HSCT is proposed as a potentially curative option that can prolong the survival time.^12^ Fei XH reported that 8 patients were treated with imatinib followed by HSCT. These patients' median OS time was 17 months.^13^ Another report showed that 17 patients were treated with imatinib combined with traditional chemotherapy and then bridged to HSCT. The five‐year OS rate was 69.3%.^10^ Furthermore, Yılmaz summarized the ongoing trials recruiting CML‐MBP patients. The management of Ph + AML can refer to CML‐MBP because of the similar clinical characteristics.^14^ We expect new agents to yield better clinical results.

The patient was diagnosed with Ph + AML rather than CML‐BP for the following reasons. She had no history of CML‐CP, and no hepatomegaly or splenomegaly was detected at diagnosis. The karyotype at diagnosis was 46, XX, t(9;22)(q34;q11)[20], with no additional clonal chromosomal abnormalities in Ph + cells. In addition, no *ABL1* kinase mutation was detected. In our study, the patient failed to achieve CR after the HA regimen. She achieved CR by the application of 5‐azacitidine, cytarabine and imatinib. The disease relapsed after imatinib was discontinued, and a short remission was obtained by imatinib and venetoclax. However, relapse occurred again and the combination of imatinib and venetoclax did not work. Previous experience has shown that HSCT contributes to sustained remission and better survival. However, the patient had a history of modified radical mastectomy, urinary tract infection, UCTD, and lacunar infarction, and she was in an anxiety state. The hematopoietic cell transplantation‐specific comorbidity index (HSC‐CI) score was evaluated at 7, and it predicted that the prognosis of HSCT would be poor.^15^ The treatment regimen was then changed to flumatinib combined with venetoclax.

Flumatinib is a second‐generation TKI independently developed and approved for the treatment of CML in China. Patients receiving flumatinib achieved significantly higher rates of responses, and faster and deeper responses without serious adverse events than those receiving imatinib, indicating that flumatinib can be an effective first‐line treatment for chronic myeloid leukemia blast crisis.^16^ In a preclinical study, the combination of TKI and venetoclax had a significant inhibitory effect in a murine CML model. Carter demonstrated increased BCL‐2 expression in bone marrow cells, particularly in Lin(−)Sca‐1(+)cKit(+) cells of inducible CML in mice. Furthermore, selective inhibition of BCL‐2, aided by TKI‐mediated MCL‐1 and BCL‐XL inhibition, markedly decreased leukemic Lin(−)Sca‐1(+)cKit(+) cell numbers and prolonged survival time in a murine CML model.^17^ Leonard demonstrated that the combination of TKIs with venetoclax is highly synergistic in decreasing cell viability and inducing apoptosis in Ph + ALL cells in vitro. They also demonstrated that dasatinib and ponatinib could overcome the resistance of venetoclax.^18^ Based on this evidence in vitro and in vivo, it is feasible and effective to combine TKIs with venetoclax in hematological malignancies.

In our case report, the patient achieved CR immediately with flumatinib and venetoclax. The patient has been in relapse‐free status for 7 months. In addition, this regimen is economically beneficial and acceptable for patients. It is also suggested that patients who cannot tolerate HSCT can be offered a treatment regimen to help achieve remission. However, more relevant clinical trials are required to further demonstrate the effectiveness of flumatinib combined with venetoclax in the future. Our results demonstrate promising efficacy and require a prospective clinical trial to further explore this treatment regimen.

## AUTHOR CONTRIBUTIONS

SMH, TT, and CLW wrote the manuscript. TMW, HYC, and YQ revised the manuscript. XDS, BRW, YYL and SSG made the figure. TTZ, BW, and SLX took care of the patient.

## FUNDING INFORMATION

This work was supported by the grants from the National Natural Science Foundation of China (Grant No. 81970138, 82270165), Translational Research Grant of NCRCH (Grant No. 2020ZKMB05), Jiangsu Province “333”project, Social Development Project of the Science and Technology Department of Jiangsu (Grant No. BE2021649), and Gusu Key Medical Talent Program (Grant No. GSWS2019007).

## CONFLICT OF INTEREST

The authors declare no competing interests.

## ETHICS STATEMENT

The patient has provided written informed consent for the publication of this case report.

## CONSENT

Written informed consent for the case to be published was obtained from the patient.

## Data Availability

The data presented in this study are available in this article.
